# Functional-genomic prioritization identifies *MED12* as a candidate RNAi vulnerability in acute myeloid leukemia: evaluation of a T22 peptide-functionalized LNP-siRNA formulation

**DOI:** 10.3389/fphar.2026.1923515

**Published:** 2026-09-23

**Authors:** Zhouchun Chen, Tianran Chai, Meichen Liu, Haigang Wu, Yangjie Li, Wenyuan Yang

**Affiliations:** 1 School of Medicine, Xinjiang University of Science and Technology, Korla, Xinjiang, China; 2 School of Life Sciences, Henan University, Kaifeng, Henan, China; 3 Department of Cancer Biomedical Science, Graduate School of Cancer Science and Policy, National Cancer Center, Goyang, Republic of Korea; 4 School of Basic Medical Science, Henan University, Kaifeng, Henan, China

**Keywords:** acute myeloid leukemia, CXCR4, functional genomics, lipid nanoparticles, *MED12*, RNA interference, siRNA, T22 peptide

## Abstract

**Background:**

Acute myeloid leukemia (AML) depends in part on transcriptional and epigenetic programs that can be difficult to target with conventional small molecules. RNA interference provides a potential approach to perturb such vulnerabilities, but target selection and intracellular siRNA delivery remain major challenges.

**Methods:**

Public dependency datasets and primary AML and normal hematopoietic expression resources were integrated to prioritize candidate RNAi vulnerabilities. A T22 peptide-functionalized lipid nanoparticle (TLNP) formulation carrying siMED12 was evaluated in OCI-AML3 cells, with RT-qPCR and CCK-8 validation in THP-1 cells. A systemic OCI-AML3 mouse model was used for exploratory assessment of survival and tolerability.

**Results:**

*MED12* ranked highest in the integrated functional-genomic prioritization framework. In OCI-AML3 and THP-1 cells, TLNP-siMED12 produced replicated reductions in *MED12* transcript abundance and cell viability across three independently initiated experiments. In OCI-AML3 cells, cellular uptake, free T22 competition, immunoblotting, apoptosis, migration and whole-well crystal violet-stained colony-like focus observations were each obtained once and are reported descriptively. In the systemic model, TLNP-siMED12 treatment was associated with prolonged survival; body weight, endpoint blood measurements and illustrative H&E images provided limited tolerability information.

**Conclusion:**

Integrated functional-genomic prioritization identified *MED12* as a candidate RNAi vulnerability in AML. TLNP-siMED12 produced reproducible transcript and viability responses in the evaluated AML models and was associated with prolonged survival in the systemic OCI-AML3 model. Hematopoietic selectivity, receptor-dependent delivery and *in vivo* target engagement remain to be established.

## 1 Introduction

Acute myeloid leukemia (AML) is a heterogeneous hematologic malignancy in which therapeutic resistance and relapse remain major clinical challenges (Döhner et al., 2015; Hackl et al., 2017; Short et al., 2018). AML-cell survival can depend on transcriptional and epigenetic programs that are difficult to modulate with conventional small molecules (Bradner et al., 2017; Roe et al., 2015). RNA interference (RNAi) offers a means to suppress difficult-to-drug intracellular candidates (Lieberman, 2018; Setten et al., 2019), but naked siRNA is rapidly degraded, enters cells inefficiently and lacks intrinsic disease-cell selectivity (Whitehead et al., 2009; Dowdy, 2017). Lipid nanoparticles (LNPs) provide a clinically relevant framework for nucleic acid delivery, although delivery to hematologic malignancies remains context dependent (Hou et al., 2021).

Large-scale functional-genomic resources can identify genes whose perturbation is associated with vulnerability in defined cancer-cell contexts (Behan et al., 2019; Dempster et al., 2021; Hahn et al., 2021; Huang et al., 2020; Pacini et al., 2024). However, dependency ranking alone cannot establish disease specificity, selectivity relative to normal cells, or a therapeutic window (Kaelin, 2017). Mediator complex subunit 12 (*MED12*) participates in transcriptional regulation through the Mediator complex (Malik and Roeder, 2010; Soutourina, 2018). Its reported role in hematopoietic stem-cell enhancer regulation establishes biological relevance to hematopoiesis but also raises concern about potential effects in normal hematopoietic cells (Aranda-Orgilles et al., 2016; Laurenti and Göttgens, 2018; Orkin and Zon, 2008). Accordingly, the present workflow combined public dependency data with primary AML, normal hematopoietic and experimental-feasibility evidence to prioritize a candidate for focused experimental evaluation rather than to infer a clinically validated therapeutic target.

CXCR4 is associated with AML-cell trafficking, bone-marrow retention and stromal interaction (Burger and Peled, 2009; Zeng et al., 2009), providing a rationale for evaluating a T22-containing delivery formulation. The tested ionizable LNP contained DLin-MC3-DMA, DOPE, cholesterol, DMG-PEG2000 and DSPE-PEG2000-T22. Within the formulation, DLin-MC3-DMA served as the ionizable lipid for siRNA association and intracellular delivery, DOPE as a helper phospholipid, cholesterol to support membrane packing and particle stability, DMG-PEG2000 for colloidal stabilization, and DSPE-PEG2000-T22 for T22 display (Wan et al., 2014; Hou et al., 2021; Yang et al., 2024). T22 was included on the basis of its reported affinity for CXCR4. Recent work has also demonstrated peptide-based siRNA nanoassembly for AML therapy, using a distinct carrier-free architecture, therapeutic cargo and targeting strategy (Yang et al., 2025).

We therefore asked whether an archived multi-component functional-genomic ranking, interpreted alongside primary AML and normal hematopoietic expression data, could prioritize a tractable AML RNAi candidate and whether TLNP-siMED12 could produce measurable molecular and phenotypic responses in *MED12*-dependent AML models.

## 2 Materials and methods

### 2.1 Public functional-genomic target prioritization

Public functional-genomic datasets were integrated to identify candidate RNAi vulnerabilities in AML. DepMap Public 26Q1 was used as the primary CRISPR/Chronos dependency dataset, with DepMap Public 25Q3 used to assess release robustness. Cell-line annotations were used to classify AML, pan-blood cancer and non-blood cancer models. Metric-specific denominators were reported because not all annotated models had evaluable values for every data layer.

The prioritization framework integrated AML dependency strength, AML-versus-non-blood selectivity, pan-blood dependency context, clinical relevance, mechanistic interpretability, siRNA feasibility, and predefined expression-related context and risk components. BeatAML, TCGA-LAML, healthy CD34-positive and DICE analyses (Schmiedel et al., 2018) were used as supporting expression context and did not alter the archived quantitative ranking. *MED12* was advanced to focused experimental testing because it ranked first in the archived quantitative framework. Detailed metric definitions, component rules, score directions, thresholds, missing-data handling, the complete candidate-ranking output, and the documented relationship between quantitative ranking and experimental follow-up are provided in Supplementary Tables S1–S3. Supplementary Figure S1 summarizes the data provenance, scoring structure and candidate-ordering procedure.

### 2.2 Exploratory BeatAML context analyses

Exploratory BeatAML context analyses were performed after candidate prioritization and did not contribute to or modify the archived candidate ranking. For the co-expression analysis, Spearman rank correlations were calculated between *MED12* expression and a curated set of AML-associated transcriptional, chromatin-regulatory, proliferation, survival and differentiation-related genes using paired expression measurements from the retained BeatAML normalized-expression matrix. The analysis was restricted to selected genes and was not intended as an unbiased transcriptome-wide discovery analysis. For the *MED12*-high versus *MED12*-low analysis, samples were dichotomized at the cohort median *MED12* expression. Genes showing the strongest directional differences between the two groups were evaluated against curated AML-relevant gene sets; enrichment was summarized using overlap counts, odds ratios and nominal P values. These analyses were used for biological context and were not interpreted as evidence of direct *MED12* regulation or causal downstream signaling. The results are shown in Supplementary Figure S2.

For clinical-context analyses, *MED12* expression was summarized descriptively within available BeatAML ELN 2017, *FLT3-ITD* and *NPM1* annotation groups using the samples with evaluable expression and corresponding clinical annotations. For drug-response context, Spearman rank correlations were calculated between *MED12* expression and available *ex vivo* drug-response area-under-the-curve (AUC) values on a compound-specific complete-case basis; therefore, the evaluable sample number varied among compounds. Lower AUC represents greater *ex vivo* drug sensitivity, whereas higher AUC represents lower sensitivity. The displayed compounds were selected to illustrate the strongest or biologically relevant associations and were not used to define *MED12* as a predictive biomarker or direct pharmacological target. The displayed P values were treated as nominal and no confirmatory inference was based on these analyses. The clinical/subtype and drug-response analyses are shown in Supplementary Figure S3.

### 2.3 Cell culture

The human AML cell line OCI-AML3 was obtained from the Leibniz Institute DSMZ–German Collection of Microorganisms and Cell Cultures GmbH (Braunschweig, Germany; catalogue no. ACC 582; RRID:CVCL_1844). The human acute monocytic leukemia cell line THP-1 was obtained from the American Type Culture Collection (ATCC, Manassas, VA, USA; catalogue no. TIB-202; RRID:CVCL_0006).

Repository documentation reported reference STR profiles supporting the identities of the deposited cultures and mycoplasma-negative status at the time of release. Independent STR authentication and study-specific *mycoplasma* testing were not performed for the cultures used in this study. Cell morphology and suspension-growth characteristics were monitored routinely.

OCI-AML3 cells were cultured in α-minimum essential medium containing ribonucleosides and deoxyribonucleosides, supplemented with 20% fetal bovine serum and 1% penicillin–streptomycin. THP-1 cells were cultured in RPMI-1640 medium supplemented with 10% fetal bovine serum and 1% penicillin–streptomycin. Both cell lines were maintained in suspension at 37 °C in a humidified atmosphere containing 5% CO_2_, passaged every 2–3 days and maintained at low passage numbers after recovery. THP-1 was used as an additional genetically and cellularly distinct *MED12*-dependent AML model for replicated RT-qPCR and CCK-8 experiments.

### 2.4 Preparation of DSPE-PEG2000-T22

The T22 peptide (QYAOBIO, ChinaPeptides Co., Ltd., China; amino-acid sequence RRWCYRKCYKGYCYRKCR; purity >95%) was coupled to DSPE-PEG (2000) Carboxy NHS (Avanti Research/Avanti Polar Lipids, LLC; catalogue no. A88138, legacy catalogue no. 880138; purity >85%) through NHS ester-mediated amine coupling. T22 and DSPE-PEG (2000) Carboxy NHS were dissolved in dimethyl sulfoxide and mixed at a molar ratio of 1.2:1 in the presence of triethylamine under mildly alkaline conditions. The reaction proceeded overnight at room temperature with gentle stirring.

The reaction mixture was dialyzed against purified water for 2 days using a 3.5-kDa molecular-weight-cutoff (MWCO) membrane, lyophilized and stored at −20 °C. BCA-based quantification after dialysis indicated approximately 98% T22 recovery/association with the purified product.

### 2.5 Preparation of T22 peptide-functionalized LNP-siRNA

T22 peptide-functionalized lipid nanoparticles carrying siRNA were termed TLNP-siRNA. The formulation consisted of DLin-MC3-DMA (Avanti Research/Avanti Polar Lipids, catalogue no. A89906, legacy no. 890906; purity >99%), DOPE (A80725, legacy 850725; purity >99%), plant-derived cholesterol (A80100, legacy 700100; purity >99%), DMG-PEG2000 (A80151, legacy 880151; purity >99%) and in-house-prepared DSPE-PEG2000-T22. The lipid molar ratio was 50:10:38:1.5:0.5, corresponding to 50.0, 10.0, 38.0, 1.5 and 0.5 mol%, respectively. The total PEG-lipid content was 2.0 mol%.

The N/P ratio was fixed at 6:1 and was defined as the molar ratio of protonatable amine nitrogen groups contributed by DLin-MC3-DMA to phosphate groups contributed by the siRNA duplex. Lipids were dissolved in ethanol and siRNA was dissolved in citrate buffer at pH 4.0. The phases were rapidly mixed at an organic-to-aqueous volume ratio of 1:3, diluted with PBS and dialyzed to reduce residual ethanol and unassociated siRNA. Nanoparticles were stored at 4 °C and used within 48 h. Because siRNA encapsulation efficiency was not directly quantified, siRNA-equivalent refers throughout this study to the nominal amount of siRNA initially introduced during nanoparticle preparation rather than to an experimentally measured encapsulated-siRNA concentration or dose. For *in vitro* experiments, the indicated formulation was used at 50 nM siRNA-equivalent unless otherwise stated.

### 2.6 Characterization of TLNP-siRNA

Particle hydrodynamic diameter, size distribution and PDI were measured using a Zetasizer Nano ZS (model ZEN3600; Malvern Panalytical), and zeta potential was measured using the same instrument after dilution in deionized water. Data acquisition and analysis were performed using Zetasizer Software version 7.13. Nanoparticle morphology was examined after negative staining with phosphotungstic acid using a JEM-2100 transmission electron microscope (JEOL, Japan).

Short-term stability was assessed by monitoring particle size and PDI during storage at 4 °C and after incubation in DMEM containing 10% (v/v) FBS at 37 °C. Gel retardation and detergent-mediated release were assessed qualitatively. For detergent-mediated release, TLNP-siRNA was incubated with 1% (v/v) Triton X-100 (Sigma-Aldrich/Merck; catalogue no. T8787) for 30 min at room temperature to disrupt the LNP structure and release siRNA before electrophoresis.

### 2.7 siRNA sequences and reagents

All siRNA oligonucleotides were synthesized by GenePharma Co., Ltd. (Shanghai, China). Two non-overlapping siRNAs targeting human *MED12* were evaluated during initial sequence screening. The siRNA sequences used were siMED12-1, 5′-UUC UCU GCA AUA AUG CUG CUG-3′, and siMED12-2, 5′-UAU UAC AAC GUA AUU UCU CUG-3′, as previously reported (Feng et al., 2025). The scrambled negative-control siRNA, siScramble, was 5′-UUC UCC GAA CGU GUC ACG UTT-3′. Cy5-modified siScramble from the same supplier was used only for qualitative cellular uptake imaging. siMED12-1 was selected for the subsequent TLNP-based experiments on the basis of its greater transcript-level knockdown during the initial sequence screening. siMED12-2 was additionally evaluated in the Lipofectamine 2000-mediated cell viability experiment.

### 2.8 Cellular uptake assay

For qualitative uptake imaging, OCI-AML3 cells were exposed to free Cy5-modified siScramble or TLNP loaded with Cy5-modified siScramble at 50 nM siRNA-equivalent for 6 h. Cells were washed, stained with DAPI and imaged using a ZEISS LSM 880 confocal laser-scanning microscope. Image acquisition and initial processing were performed using ZEN software version 2.3, black edition, with identical imaging settings across groups.

In the single qualitative free T22 competition experiment shown in Figure 1D, the groups were Free siRNA, TLNP-siRNA + Free T22, and TLNP-siRNA. Cells in the TLNP-siRNA + Free T22 group were pre-blocked with free T22 (1 μM, 37 °C, 1 h) before exposure to TLNP-siRNA (50 nM siRNA-equivalent). The competition experiment was performed once and assessed whether excess free T22 altered the TLNP-associated uptake signal.

**FIGURE 1:**
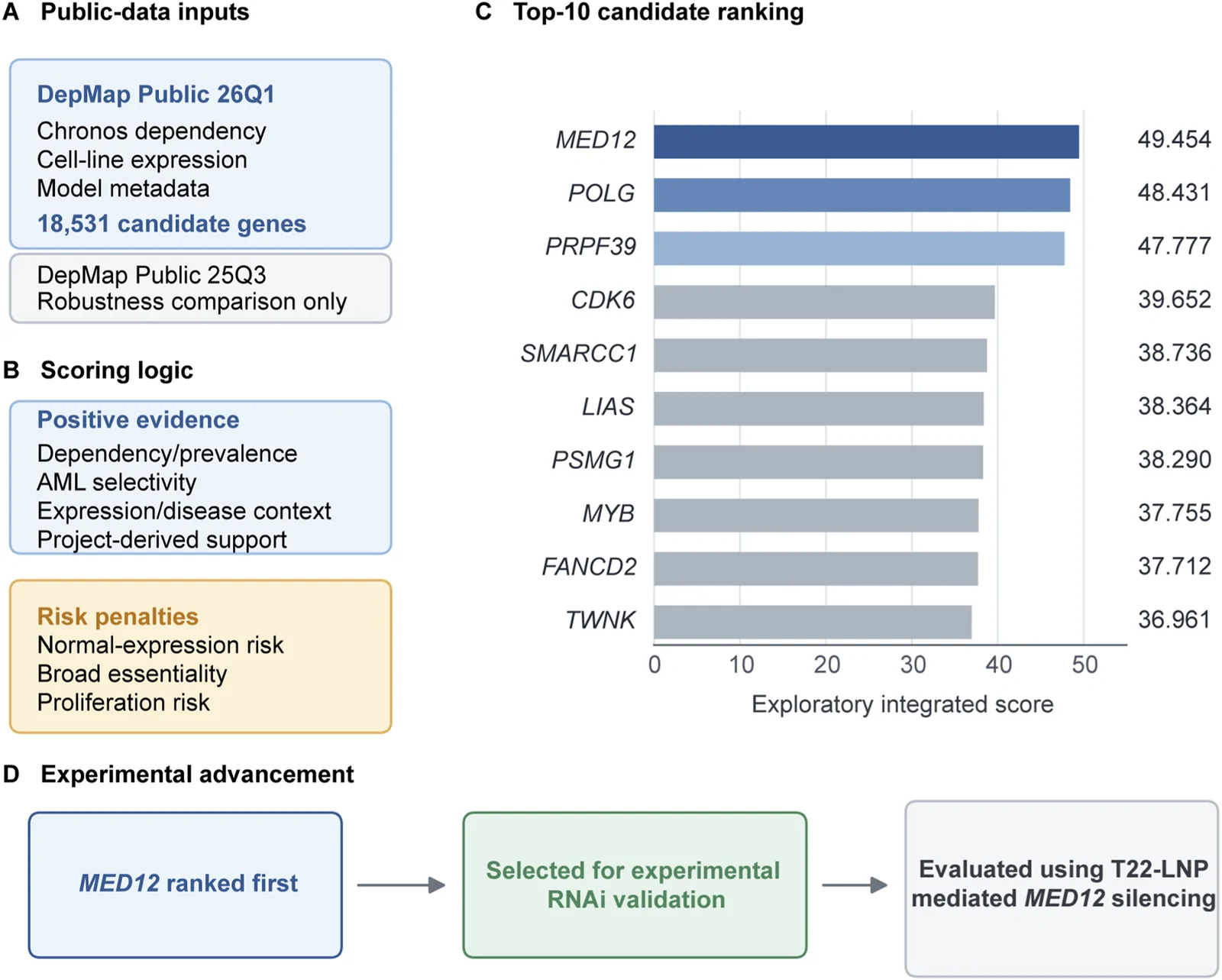
Public functional-genomic prioritization identifies *MED12* as a focused AML RNAi candidate. **(A)** DepMap Public 26Q1 inputs used for the primary data-derived ranking components and DepMap Public 25Q3 used for release-robustness comparison. **(B)** Scoring logic combining data-derived components, project-derived numerical components and predefined risk-proxy penalties. **(C)** Top-10 candidate ranking based on the exploratory integrated score. **(D)** Experimental advancement of *MED12* from the ranked candidate set to RNAi validation with the T22 peptide-functionalized LNP formulation. Detailed metric definitions, score directions, thresholds, missing-data handling, the complete candidate-ranking output and the relationship between quantitative ranking and experimental follow-up are provided in Supplementary Tables S1 -S3. Additional code and supporting data are available from the corresponding author upon reasonable request.

### 2.9 Quantitative real-time PCR

OCI-AML3 and THP-1 cells were treated with PBS, TLNP-siScramble or TLNP-siMED12 at 50 nM siRNA-equivalent. Total RNA was extracted 48 h after treatment. First-strand cDNA was synthesized using the RevertAid First Strand cDNA Synthesis Kit (Thermo Fisher Scientific; catalogue no. K1622), and RT-qPCR was performed using PowerUp SYBR Green Master Mix (Applied Biosystems; catalogue no. A25742) on a QuantStudio 5 Real-Time PCR System. Data were processed using QuantStudio Design and Analysis Desktop Software version 1.5.1. Relative expression was calculated using the 2^−ΔΔCT^ method and normalized to *GAPDH*. Primer sequences were *MED12* forward 5′-GCT​GCT​CCT​CTA​ACA​ATG​GCA​C-3′, *MED12* reverse 5′-TCT​TCC​AGG​AGC​AAA​CAC​TGC​C-3′, *GAPDH* forward 5′-GGA​GCG​AGA​TCC​CTC​CAA​AAT-3′ and *GAPDH* reverse 5′-GGC​TGT​TGT​CAT​ACT​TCT​CAT​GG-3′.

For the initial siRNA-sequence screening, OCI-AML3 cells were transfected with siScramble, siMED12-1 or siMED12-2 at a final siRNA concentration of 50 nM using Lipofectamine 2000 (Thermo Fisher Scientific), and PBS was included as a reference control. *MED12* mRNA expression was measured using the same RT-qPCR workflow. The screening was performed in three independently initiated biological experiments, each comprising three technical wells. Technical wells were averaged within each experiment, and the experiment-level 2^−ΔΔCT^ value was used as the statistical unit. The comparison used for sequence selection was between siMED12-1 and siMED12-2.

For the TLNP-based experiments, RT-qPCR was performed separately in OCI-AML3 and THP-1 cells in three independently initiated biological experiments, each with three technical wells. Technical wells were averaged within each experiment, and the experiment-level 2^−ΔΔCT^ value was used for statistical analysis. TLNP-siScramble and TLNP-siMED12 were compared using an unpaired t-test in GraphPad Prism version 8.3.0. PBS was displayed as a reference control and was not included in the inferential comparison.

### 2.10 Western blotting

OCI-AML3 cells were treated with PBS, TLNP-siScramble or TLNP-siMED12 at 50 nM siRNA-equivalent and harvested 72 h after treatment. Proteins were extracted in RIPA buffer, separated by SDS-PAGE and transferred to PVDF membranes. After blocking, membranes were incubated overnight at 4 °C with rabbit recombinant monoclonal anti-MED12 (Abcam, ab300154; 1:1,000), rabbit polyclonal anti-Caspase-3 (Abcam, ab13847; 1:1,000), rabbit polyclonal anti-cleaved Caspase-3 (Abcam, ab2302; 1:1,000), mouse monoclonal anti-β-actin (Abcam, ab8226; 1:1,000) or rabbit monoclonal anti-Vinculin (Abcam, ab129002; 1:10,000), as appropriate. Protein bands were detected by enhanced chemiluminescence and imaged using an Amersham Imager 680RGB system (GE Healthcare, Japan).

Immunoblotting was performed once and no densitometric quantification or inferential statistical analysis was performed.

### 2.11 Cell viability assay

For the Lipofectamine 2000-mediated siRNA-sequence viability experiment, OCI-AML3 cells were transfected with siScramble, siMED12-1 or siMED12-2 at a final siRNA concentration of 50 nM, and PBS was included as a reference control. For the subsequent TLNP-based experiments, OCI-AML3 and THP-1 cells were treated with PBS, TLNP-siScramble or TLNP-siMED12 at 50 nM siRNA-equivalent.

For all CCK-8 experiments, cells were seeded in 96-well plates at 5 × 10^3^ cells/well. At 72 h after transfection or treatment, 10 μL of Cell Counting Kit-8 reagent (Dojindo Molecular Technologies; catalogue no. CK04) was added to each well and incubated for 2 h at 37 °C. Absorbance at 450 nm was measured using a SpectraMax i3x microplate reader (Molecular Devices, San Jose, CA, USA), and viability was normalized to the corresponding PBS control.

Each CCK-8 experiment comprised three independently initiated biological experiments, with three technical wells per condition in each experiment. Technical wells were averaged within each experiment, and the independently initiated experiment was used as the statistical unit. For the Lipofectamine 2000-mediated siRNA-sequence experiment, the indicated pairwise comparisons were analyzed using unpaired two-tailed t-tests. For the TLNP-based experiments, TLNP-siScramble and TLNP-siMED12 were compared using an unpaired t-test. PBS was displayed as a reference control and was not included in the inferential comparisons. Statistical analyses were performed using GraphPad Prism version 8.3.0.

### 2.12 Flow-cytometric apoptosis assay

OCI-AML3 cells were treated with PBS, TLNP-siScramble or TLNP-siMED12 at 50 nM siRNA-equivalent. After 72 h, cells were stained using the FITC Annexin V Apoptosis Detection Kit I (BD Biosciences/BD Pharmingen; catalogue no. 556547) for 15 min at room temperature in the dark. Data were acquired using a BD FACSCanto II flow cytometer operated with BD FACSDiva software version 8.0.3 and analyzed using FlowJo version 10.8.1. Debris was excluded by forward- and side-scatter gating, and total apoptotic cells were defined as the sum of early- and late-apoptotic populations.

### 2.13 Transwell migration assay

OCI-AML3 cells were pretreated with PBS or TLNP-siMED12 at 50 nM siRNA-equivalent for 48 h and assessed using Transwell inserts with 8-μm pores. Medium containing 10% fetal bovine serum was used in the lower chamber as a chemoattractant, and migration proceeded for 24 h. For fluorescent cell labeling, cells were stained with Vybrant DiI Cell-Labeling Solution (Thermo Fisher Scientific/Invitrogen; catalogue no. V22885) at a final concentration of 5 μM for 20 min at 37 °C in the dark, followed by washing before imaging.

### 2.14 Crystal violet-stained colony-like focus assay

OCI-AML3 cells were treated with PBS or TLNP-siMED12 at 50 nM siRNA-equivalent. After treatment, equal numbers of viable cells were transferred to culture plates and maintained until visible cell aggregates or colony-like foci formed. The cultures were subsequently fixed, stained with crystal violet and imaged as whole wells.

### 2.15 Systemic OCI-AML3 AML xenograft model and *in vivo* treatment

Male BALB/c nude mice carrying the *Foxn1* ^*nu*^ mutation (CAnN.Cg-*Foxn1* ^*nu*^/Crl; strain code 401), 6–8 weeks old and weighing 18–22 g at receipt, were obtained from Beijing Vital River Laboratory Animal Technology Co., Ltd. (Beijing, China). No irradiation, chemotherapy or other myeloablative preconditioning was used before inoculation. OCI-AML3 cells in logarithmic growth phase were washed and resuspended in sterile PBS; each mouse received 5 × 10^6^ viable cells intravenously in 100 μL PBS. No anesthesia was used for tail-vein inoculation or intravenous administration of the formulations.

Treatment began on day 7 after inoculation. Mice were assigned by simple random allocation to PBS, TLNP-siScramble and TLNP-siMED12 groups (n = 5/group). The specific randomization procedure was not documented. As this was an exploratory study, no formal *a priori* sample-size calculation was performed, and five mice were included per group based on practical feasibility. TLNP-siScramble and TLNP-siMED12 were administered by tail-vein injection at 1 mg/kg siRNA-equivalent in 100 μL per mouse on days 7, 10, 13, 16, 19 and 22; PBS was administered by the same route and schedule. The 1 mg/kg dose was selected with reference to preclinical myeloid-leukemia studies that used systemic siRNA-nanoparticle regimens at this dose (Ansari et al., 2024; Issa et al., 2023). The formulations and dosing schedules in those studies differed from those used here, and no dose-ranging or optimization experiment was performed. Treatment administration was not blinded. Data analysis was performed under coded group labels, and treatment identities were revealed after completion of the analyses.

Body weight was measured every 2 days. Each time point was calculated from animals alive and measured at that time, with no post-death imputation. All mice were included in survival analysis, with no exclusions and no censoring. Humane endpoints included body-weight loss >20%, severe lethargy, impaired mobility, hind-limb paralysis, neurological symptoms, respiratory distress, inability to access food or water, or moribund status. Mice reaching a humane endpoint were euthanized and counted as survival events. At the predefined humane endpoint or experimental endpoint, mice were euthanized by CO_2_ inhalation using a gradual-fill method, followed by cervical dislocation to confirm death. All animal procedures were approved by the Animal Ethics Committee of Henan University (approval no. HUSOM2023-161).

### 2.16 Serum chemistry, blood counts, and histological analysis

At the experimental endpoint, serum chemistry and blood counts were measured only in the PBS and TLNP-siMED12 groups (n = 3 mice/group). Serum chemistry included alanine aminotransferase, aspartate aminotransferase, albumin, urea, creatinine and uric acid and was measured using a Chemray 240 automated chemistry analyzer (Rayto, China). White blood cells, red blood cells and platelets were measured using a BC-2800Vet automated hematology analyzer (Mindray, China).

Heart, lung, spleen, liver and kidney were fixed in 4% paraformaldehyde, embedded in paraffin, sectioned and stained with hematoxylin and eosin. Images were acquired using an IX73 inverted microscope (Olympus, Japan). One section from one mouse per group (n = 1/group) was reviewed under coded group labels. No predefined formal pathology scoring system was applied.

### 2.17 Quantification and statistical analysis

RT-qPCR and CCK-8 data, including the Lipofectamine 2000-mediated siRNA-sequence experiments, are presented as mean ± SD from three independently initiated biological experiments. Each experiment included three technical wells, which were averaged before analysis; the independently initiated experiment was the statistical unit. RT-qPCR analyses were performed on experiment-level 2^−ΔΔCT^ relative-expression values. For the Lipofectamine 2000-mediated siRNA-sequence experiments shown in Supplementary Figure S4, the indicated pairwise comparisons were performed using unpaired two-tailed t-tests. For the TLNP-based RT-qPCR and CCK-8 experiments, TLNP-siScramble and TLNP-siMED12 were compared using an unpaired t-test. PBS was displayed as a reference control where indicated and was not included in the corresponding inferential comparisons. P < 0.05 was considered statistically significant. Body-weight trajectories were analyzed using two-way ANOVA followed by Sidak’s multiple-comparisons test, and survival between TLNP-siMED12 and PBS was compared using the log-rank (Mantel–Cox) test. Cellular uptake, free T22 competition, immunoblotting, Annexin V/PI, migration and the crystal violet-stained colony-like focus assay were each performed once and are presented descriptively without inferential statistics.

Individual animals were the observational unit for animal endpoints. Survival analysis included n = 5 mice/group; endpoint serum chemistry and blood counts included PBS and TLNP-siMED12 groups only (n = 3 mice/group); H&E images represent n = 1 mouse/group for PBS, TLNP-siScramble and TLNP-siMED12. All statistical analyses were performed using GraphPad Prism version 8.3.0.

## 3 Results

### 3.1 Public functional-genomic prioritization identified *MED12* as a focused AML RNAi candidate

The integrated analysis ranked candidate genes across AML dependency, pan-blood and non-blood dependency profiles, primary AML expression, normal hematopoietic expression and feasibility layers (Figures 1A,B). *MED12* ranked first in the archived integrated quantitative framework and was therefore advanced for focused experimental testing (Figures 1C,D). Comparator genes were retained as contextual and risk-reference candidates rather than expanded into additional wet-lab targets. The complete ranking output, metric definitions, component rules, and documented relationship between quantitative ranking and experimental follow-up are provided in Supplementary Tables S1–S3.

### 3.2 *MED12* showed consistent AML dependency and primary AML expression

*MED12* showed a consistent AML dependency direction in DepMap Public 26Q1 and DepMap 25Q3 analyses (Figure 2A). Primary AML expression summaries from BeatAML and TCGA-LAML confirmed *MED12* expression across both cohorts (Figures 2B,C). Together, these data placed *MED12* among the experimentally tractable candidates identified by the prioritization workflow. The integrated evidence also informed selection of the AML models used for downstream experiments (Figure 2D).

**FIGURE 2:**
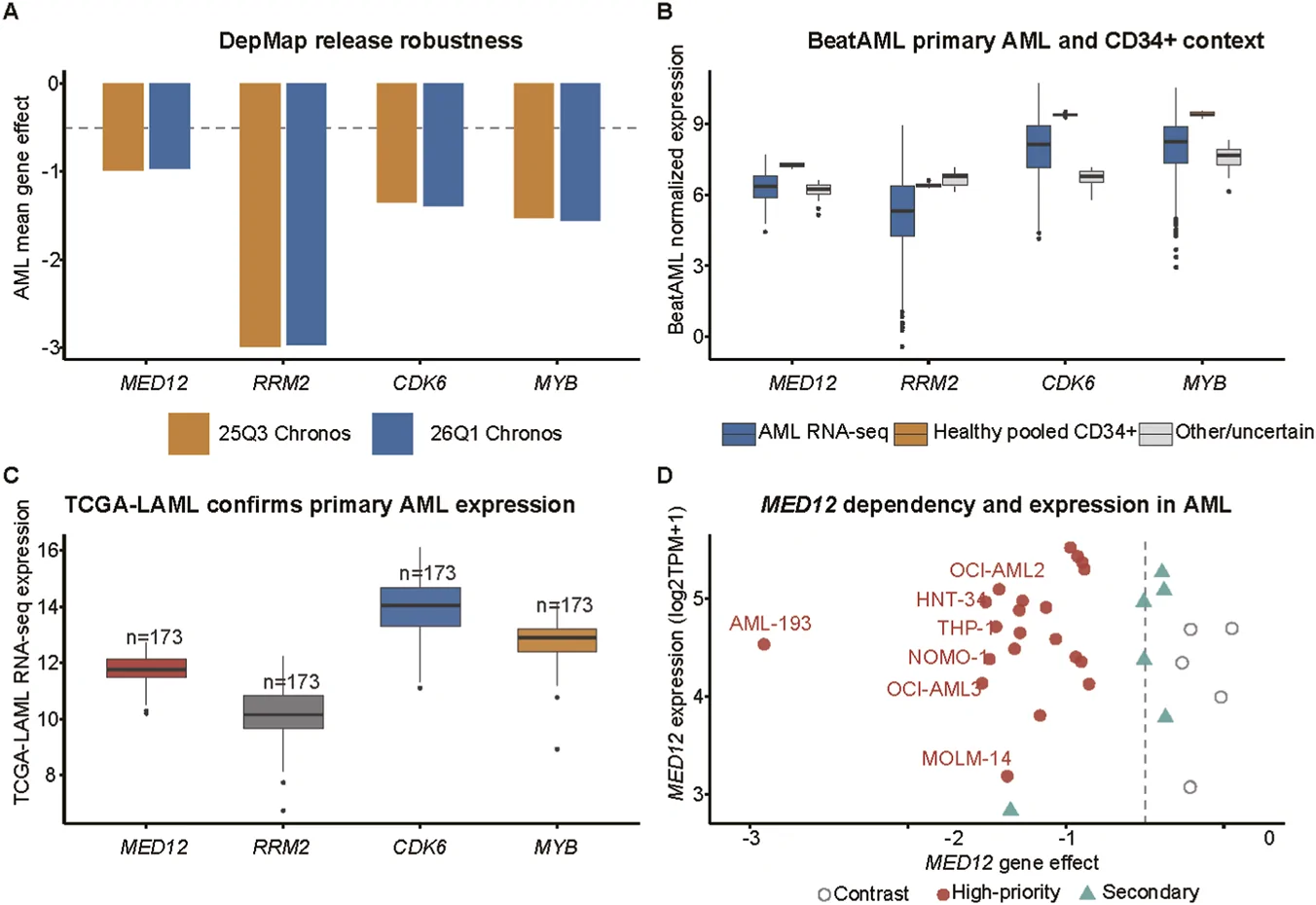
*MED12* dependency and primary AML expression context. **(A)** DepMap Public 26Q1 and 25Q3 analyses showed a consistent MED12 dependency direction in AML models. **(B,C)** BeatAML and TCGA-LAML summaries showed *MED12* expression in primary AML cohorts. **(D)** Integrated dependency and expression evidence informed selection of the AML cell models used for subsequent testing.

### 3.3 Normal hematopoietic expression defined the major boundary for *MED12* therapeutic interpretation

Public normal hematopoietic and immune-cell datasets showed that *MED12* is detectable across normal hematopoietic and immune compartments (Figures 3A–C). This expression pattern underscores the need to evaluate hematopoietic selectivity directly in future studies (Figure 3D). OCI-AML3 and THP-1 were used as MED12-dependent AML models for subsequent validation.

**FIGURE 3:**
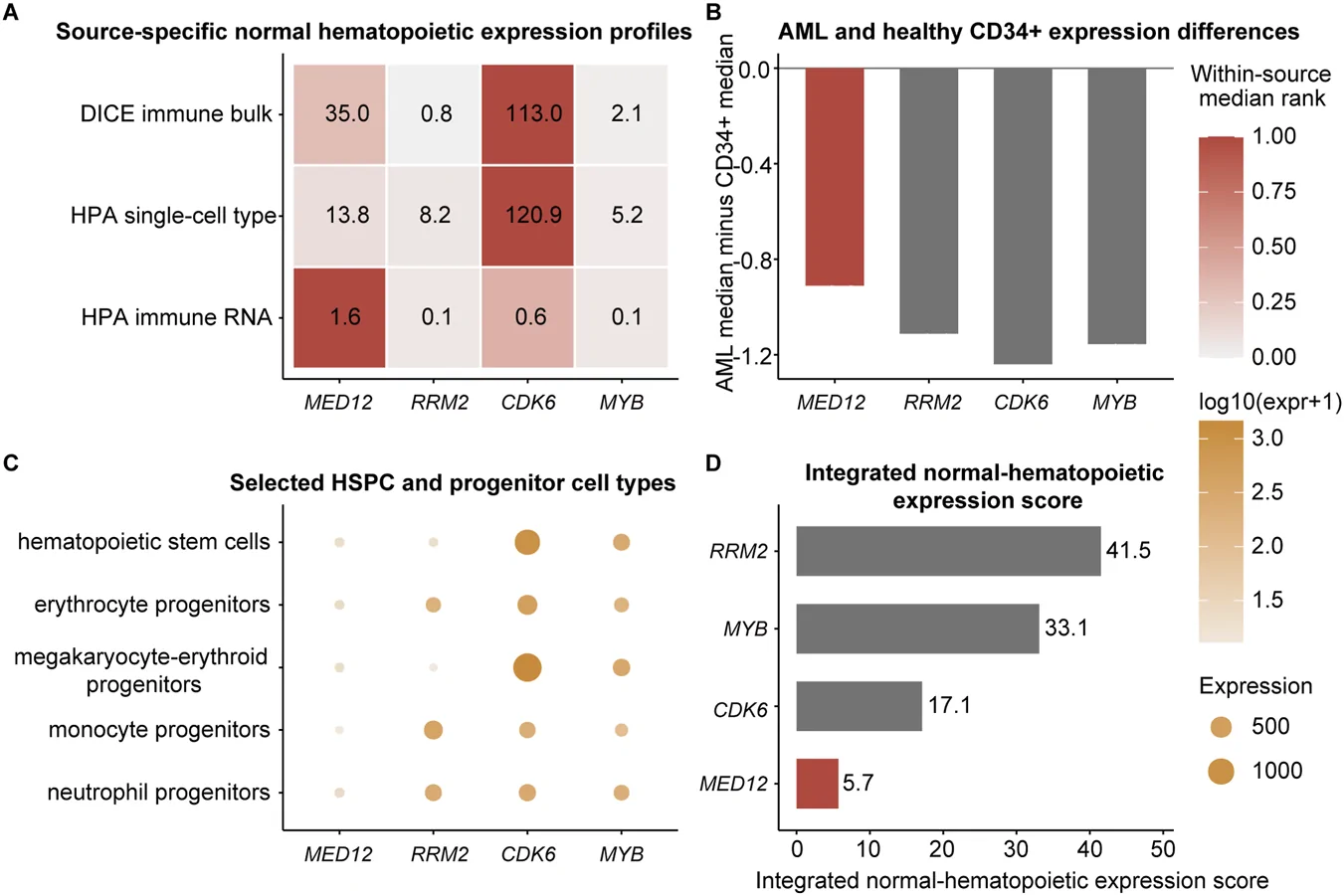
Normal hematopoietic expression context for *MED12*. **(A -C)** CD34-positive control, DICE immune-cell, Human Protein Atlas immune-RNA and Human Protein Atlas single-cell analyses showed *MED12* expression in normal hematopoietic and immune contexts. **(D)** Normal hematopoietic *MED12* expression defines an important boundary for subsequent evaluation of hematopoietic selectivity.

### 3.4 T22 peptide-functionalized LNPs showed cellular uptake in OCI-AML3 cells

TLNP-siRNA was formulated using DLin-MC3-DMA, DOPE, cholesterol, DMG-PEG2000 and DSPE-PEG2000-T22. Dynamic light scattering showed a representative particle size of 122.4 nm with a PDI of 0.234 (Figure 4A). Zeta-potential analysis defined the displayed surface-charge profile (Figure 4B), and transmission electron microscopy confirmed nanoscale particle morphology (Figure 4C). Supplementary Figure S5 shows short-term stability at 4 °C and after incubation in DMEM containing 10% (v/v) FBS at 37 °C. The qualitative gel assay used 1% (v/v) Triton X-100 for 30 min at room temperature to disrupt TLNP-siRNA and release siRNA.

**FIGURE 4:**
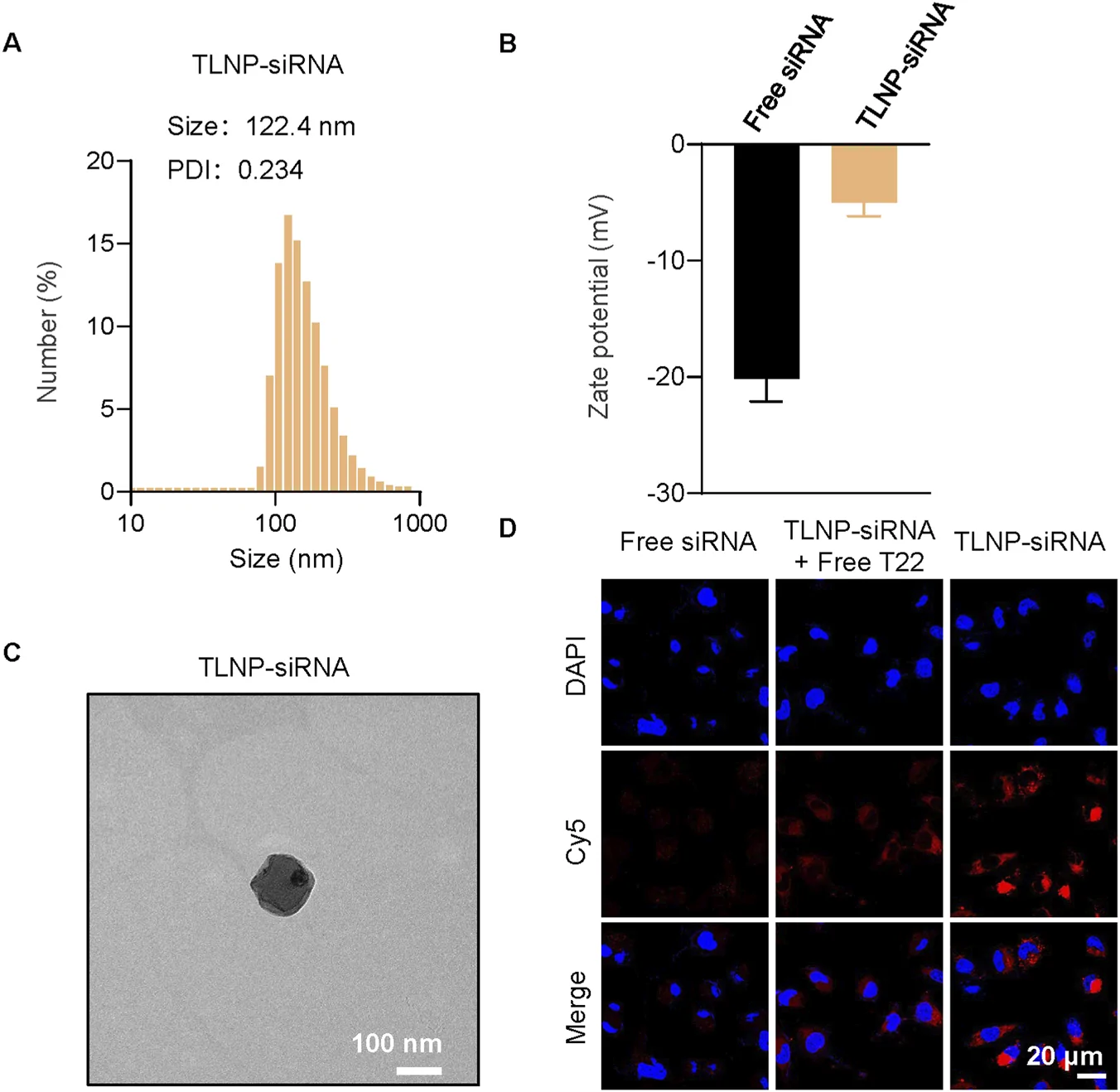
Characterization and qualitative cellular uptake of TLNP-siRNA in OCI-AML3 cells. **(A)** Dynamic light scattering showed a representative particle size of 122.4 nm with a PDI of 0.234. **(B)** Zeta-potential analysis characterized the surface-charge profile. **(C)** Transmission electron microscopy showed nanoscale particle morphology. **(D)** The uptake reagent was Cy5-modified siScramble. For the single qualitative free T22 competition experiment, the groups were Free siRNA, TLNP-siRNA + Free T22, and TLNP-siRNA. Cells in the TLNP-siRNA + Free T22 group were pre-blocked with free T22 (1 μM, 37 °C, 1 h) before exposure to TLNP-siRNA (50 nM siRNA-equivalent). Images are from one experiment and were not subjected to inferential statistical analysis.

The Cy5 uptake and free T22 competition observations were obtained once using Cy5-modified siScramble. For Figure 4D, the groups were Free siRNA, TLNP-siRNA + Free T22, and TLNP-siRNA; cells in the TLNP-siRNA + Free T22 group were pre-blocked with free T22 (1 μM, 37 °C, 1 h) before TLNP-siRNA exposure (50 nM siRNA-equivalent). Free T22 pre-blocking reduced the displayed TLNP-associated fluorescence signal, consistent with competition involving T22.

### 3.5 MED12-targeting siRNA sequence evaluation and TLNP-based validation of transcript reduction and cell viability

Before the TLNP-based experiments, two non-overlapping *MED12*-targeting siRNAs were evaluated by Lipofectamine 2000-mediated transfection in OCI-AML3 cells. Across three independently initiated biological experiments, siMED12-1 reduced *MED12* mRNA relative to siScramble (P < 0.0001) and produced a greater transcript reduction than siMED12-2 (P = 0.0084; Supplementary Figure S4A). In the corresponding CCK-8 sequence-validation experiment, siMED12-2 also showed lower mean normalized cell viability than siScramble, while siMED12-1 produced a greater viability reduction than siMED12-2 (P = 0.0011; Supplementary Figure S4B). The sequence evaluation therefore included both transcript and viability readouts. Based on its greater transcript-level knockdown, siMED12-1 was selected for the subsequent TLNP-based experiments.

Using the selected siMED12-1 sequence, TLNP-siMED12 showed lower *MED12* mRNA than TLNP-siScramble in three independently initiated experiments in OCI-AML3 cells (Figure 5A) and THP-1 cells (Supplementary Figure S6A). CCK-8 analysis likewise showed lower normalized cell viability after TLNP-siMED12 treatment than after TLNP-siScramble in OCI-AML3 cells (Figure 5C) and THP-1 cells (Supplementary Figure S6B). For both readouts, technical wells were averaged within each independently initiated experiment before analysis. TLNP-siScramble and TLNP-siMED12 were compared using an unpaired t-test.

**FIGURE 5:**
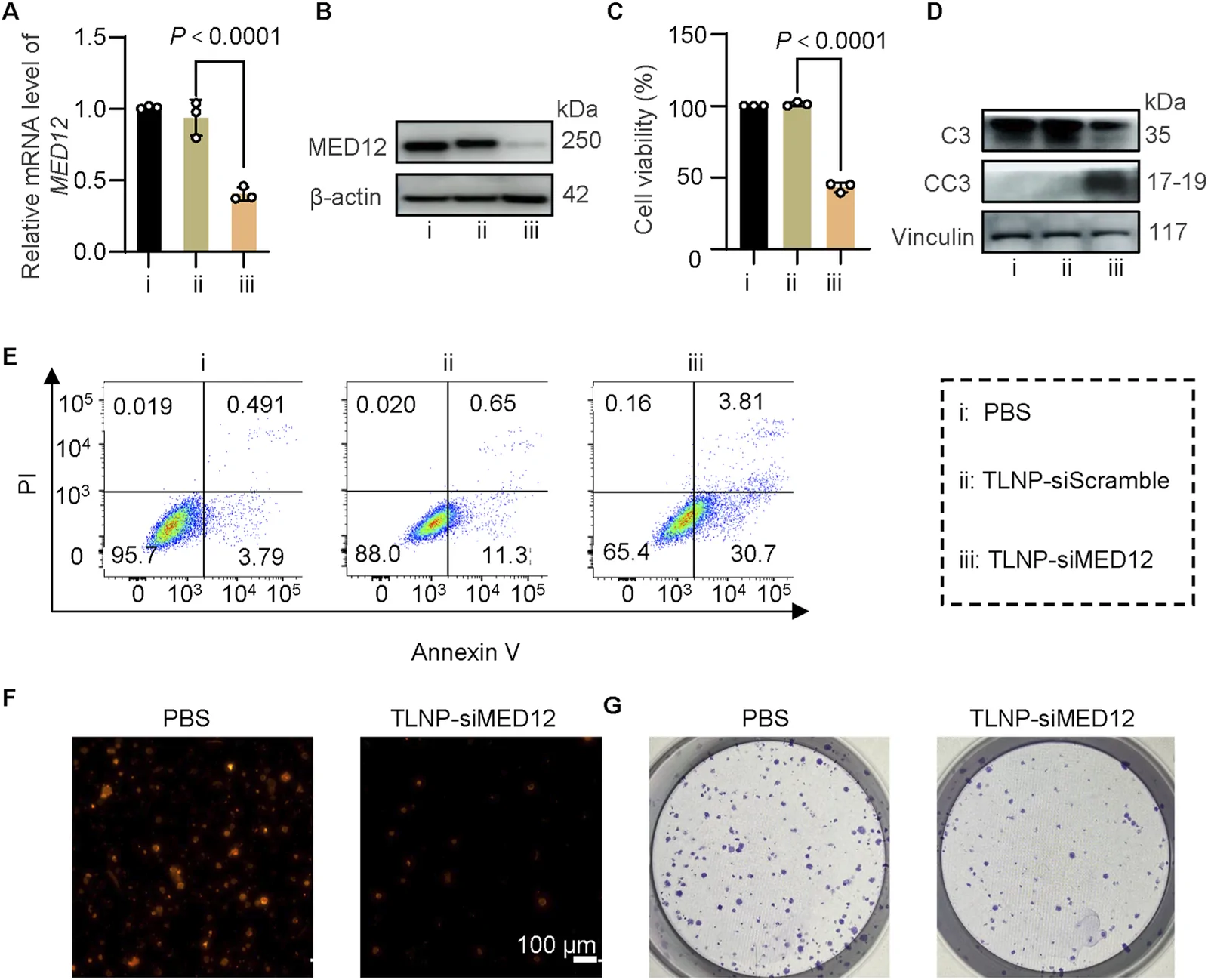
TLNP-siMED12 treatment in OCI-AML3 cells: MED12 expression, cell viability and supporting phenotypic observations. **(A)** RT-qPCR showed lower relative *MED12* mRNA expression after TLNP-siMED12 treatment (P < 0.0001). **(B)** MED12 immunoblot following treatment. **(C)** CCK-8 analysis showed lower normalized cell viability after TLNP-siMED12 treatment (P < 0.0001). For **(A,C)** data are presented as mean ± SD from three independently initiated biological experiments; three technical wells within each experiment were averaged before analysis. TLNP-siScramble and TLNP-siMED12 were compared using an unpaired t-test. For **(A)** statistical analysis was performed on experiment-level 2^^−ΔΔCt^ relative-expression values. PBS was included as a reference control and was not included in the inferential comparison. **(D)** Immunoblots for caspase-3, cleaved caspase-3 and vinculin showed visually increased cleaved caspase-3 signal after TLNP-siMED12 treatment. **(E)** Annexin V/PI analysis showed a directional increase in apoptotic-cell populations following TLNP-siMED12 treatment. **(F)** DiI-labeled Transwell images showed fewer migrated OCI-AML3 cells after TLNP-siMED12 treatment. Scale bar was 100 μm. **(G)** Whole-well crystal violet staining showed fewer visible colony-like cell foci following TLNP-siMED12 treatment. **(B,D–G)** were obtained from single experiments and are presented descriptively without inferential statistical analysis; densitometric quantification was not performed for the immunoblots. For **(A–E)** group labels are i, PBS; ii, TLNP-siScramble; and iii, TLNP-siMED12. Replicated THP-1 RT-qPCR and CCK-8 data are shown separately in Supplementary Figure S6. C3, caspase-3; CC3, cleaved caspase-3. All statistical analyses were performed using GraphPad Prism version 8.3.0.

The MED12 immunoblot in OCI-AML3 cells was obtained from a single experiment and is presented qualitatively without densitometry or inferential statistics (Figure 5B). Thus, replicated TLNP-based RT-qPCR and CCK-8 findings were obtained in both OCI-AML3 and THP-1 cells, whereas the protein-level observation was limited to a single OCI-AML3 experiment.

### 3.6 Supporting phenotypic observations following TLNP-siMED12 treatment in OCI-AML3 cells

Annexin V/PI analysis showed a directional increase in apoptotic-cell populations after TLNP-siMED12 treatment, while immunoblotting showed visually greater cleaved caspase-3 signal (Figures 5D,E). Transwell assay showed fewer migrated OCI-AML3 cells after treatment (Figure 5F), while crystal violet staining showed fewer visible colony-like foci (Figure 5G). Each of these phenotypic assays was performed once and is presented descriptively without inferential statistical analysis.

### 3.7 TLNP-siMED12 was associated with prolonged survival in a systemic OCI-AML3 AML xenograft model

Kaplan-Meier analysis included all five mice per group, with no exclusions or censoring. Survival between TLNP-siMED12 and PBS was compared using the log-rank (Mantel–Cox) test and yielded P = 0.0034 (Figure 6B). Body weight was measured every 2 days, and each time point included animals that were alive and measured at that time, with no post-death imputation. Body-weight trajectories were analyzed using two-way ANOVA followed by Sidak’s multiple-comparisons test. Treatment-group means were compared within each time point; the indicated Sidak-adjusted comparison yielded P < 0.0001 (Figure 6A).

**FIGURE 6:**
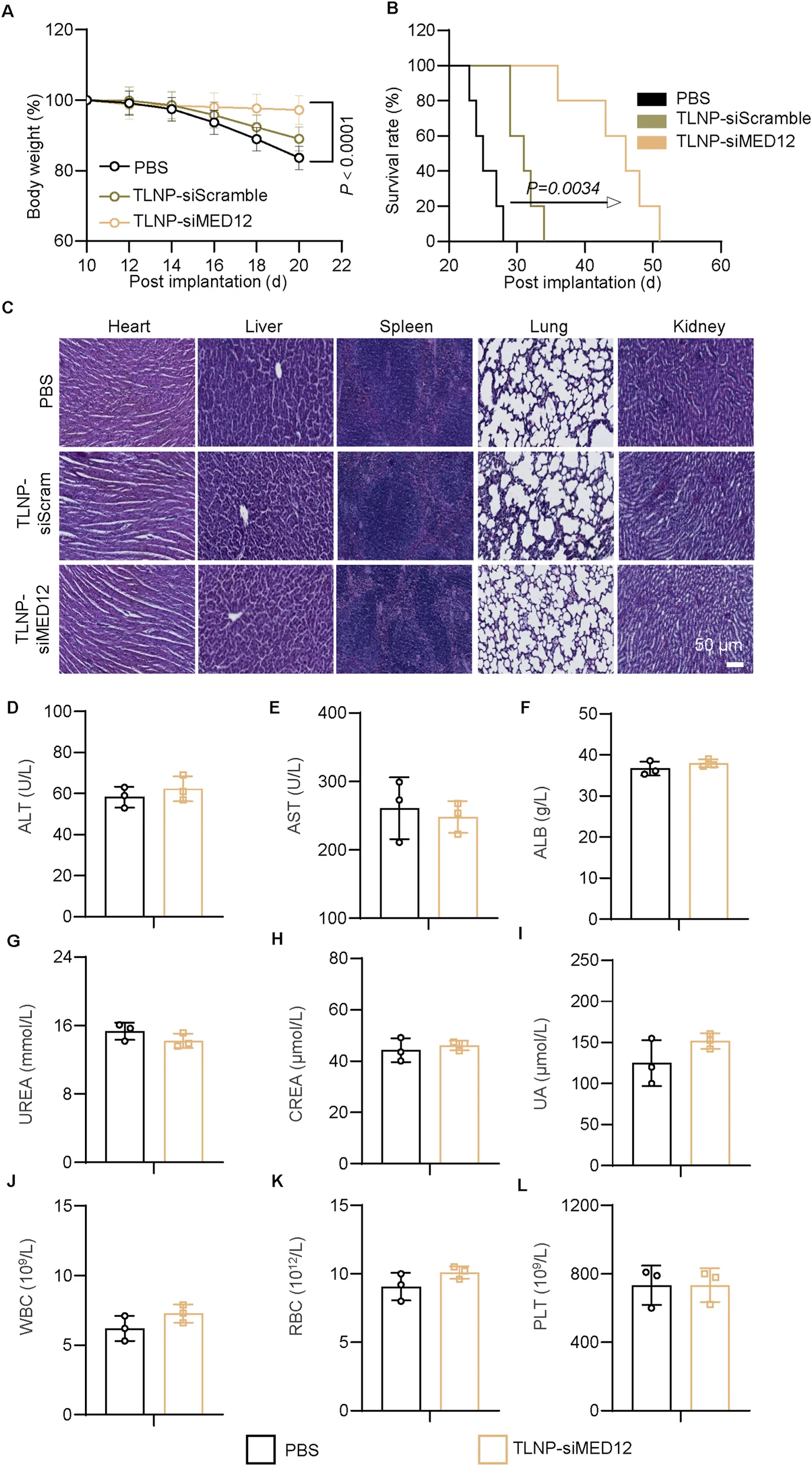
Survival and selected tolerability observations in the systemic OCI-AML3 AML model. **(A)** Body weight was measured every 2 days; each time point includes animals alive and measured at that time, without post-death imputation. Body-weight trajectories were analyzed using two-way ANOVA followed by Sidak’s multiple-comparisons test. PBS and TLNP-siMED12 group means were compared within each time point using Sidak-adjusted multiple comparisons; the indicated comparison yielded P < 0.0001. **(B)** Kaplan–Meier survival includes n = 5 mice/group, with no exclusions and no censoring; humane-endpoint deaths were recorded as events. Survival between TLNP-siMED12 and PBS was compared using the log-rank (Mantel–Cox) test (P = 0.0034). Treatment administration was not blinded; data analysis was performed under coded group labels. **(C)** H&E sections represent n = 1 mouse/group for PBS, TLNP-siScramble and TLNP-siMED12 and are presented as illustrative morphology reviewed under coded group labels without formal pathology scoring. **(D–L)** Serum chemistry and blood-count measurements were obtained only from the PBS and TLNP-siMED12 groups (n = 3 mice/group). Individual points represent individual animals, and bars indicate mean ± SD.

Serum chemistry and blood counts were obtained only from the PBS and TLNP-siMED12 groups (n = 3 mice per group; Figures 6D–L). H&E sections from n = 1 mouse per group for PBS, TLNP-siScramble and TLNP-siMED12 were reviewed under coded group labels and are presented as illustrative morphology without formal pathology scoring (Figure 6C). The blood, serum-chemistry and histological data provide only limited tolerability information.

## 4 Discussion

This study combined public functional-genomic prioritization with focused experimental evaluation of a T22 peptide-functionalized LNP-siRNA formulation to investigate *MED12* as a candidate RNAi vulnerability in AML. *MED12* was prioritized on the basis of AML dependency, primary AML expression and experimental feasibility, while normal hematopoietic expression constrained its therapeutic interpretation. The study therefore connects functional-genomic candidate prioritization with experimental RNAi testing in tractable AML models.

Evaluation of two non-overlapping *MED12*-targeting siRNAs in OCI-AML3 cells included both transcript and viability readouts. siMED12-1 produced the greater transcript-level knockdown and was therefore selected for the subsequent TLNP-based experiments. Using this sequence, replicated RT-qPCR and CCK-8 experiments in OCI-AML3 and THP-1 showed concordant reductions in *MED12* transcript abundance and cell viability. The replicated cross-model evidence in this study was therefore confined to *MED12* transcript reduction and reduced cell viability.

*MED12* is a core component of the Mediator transcriptional regulatory machinery, and its perturbation may therefore have broad transcriptional consequences. In the selected-gene BeatAML context analysis, *MED12* expression was positively associated with transcriptional and proliferation-related genes including *MYB*, *GATA2* and *CDK6*, whereas several myeloid differentiation-associated genes showed inverse relationships. The *MED12*-high versus *MED12*-low analysis likewise indicated enrichment of an AML stemness-related transcriptional context together with reduced representation of selected myeloid-differentiation genes. These associations are consistent with higher *MED12* expression occurring in a more immature AML transcriptional state and provide biological context for the viability findings without establishing a downstream mechanism. The apoptosis, migration, immunoblotting and colony-like focus observations, however, were obtained only in OCI-AML3 cells and were each based on a single experiment. Accordingly, both these phenotypic observations and the BeatAML associations should be regarded as hypothesis-generating rather than as evidence of a causal pathway or a generalizable phenotype across AML models.

The T22 peptide-functionalized LNP provided a formulation for siMED12 delivery and testing in OCI-AML3 cells. This approach is conceptually related to recent peptide-based siRNA delivery strategies explored in AML, including a carrier-free peptide–daunorubicin–siRNA nanoassembly targeting *LILRB4*, while differing substantially in formulation architecture, cargo and targeting design (Yang et al., 2025). Particle size, surface charge, morphology, short-term stability and qualitative siRNA-retention/release assays characterized the formulation used in subsequent cellular experiments. Free T22 pre-blocking reduced the TLNP-associated fluorescence signal, consistent with competition involving T22.

In the systemic OCI-AML3 model, TLNP-siMED12 treatment was associated with prolonged survival relative to PBS. Endpoint blood counts, serum chemistry and H&E examination provided limited tolerability information rather than a comprehensive safety assessment.

Several limitations constrain the interpretation of these findings. Although sequence-level validation was extended to cell viability using the second non-overlapping *MED12*-targeting siRNA, siMED12-2, the apoptosis, migration, immunoblotting and colony-like growth observations were obtained only with the selected siMED12-1 sequence in OCI-AML3 cells. THP-1 validation was limited to replicated RT-qPCR and CCK-8 experiments; these additional phenotypes therefore lack both sequence-level and cross-model replication, and no rescue experiment was performed. Both OCI-AML3 and THP-1 represent *MED12*-dependent AML models, and neither a weakly *MED12*-dependent AML model nor a nonmalignant hematopoietic comparator was directly tested. The targeting experiments did not establish CXCR4-dependent delivery, and the *in vivo* study did not quantify leukemia burden or target engagement. Future studies should confirm AML engraftment before treatment, quantify leukemia burden and biodistribution, and assess *MED12* knockdown in recovered leukemia cells. Broader AML-model validation, dose/schedule optimization, and more comprehensive hematopoietic and histopathological safety assessment will also be required. Whether baseline *MED12* expression predicts response to TLNP-siMED12 should be tested prospectively rather than inferred from the exploratory expression analyses.

## 5 Conclusion

Functional-genomic prioritization identified *MED12* as a candidate RNAi vulnerability in AML suitable for focused experimental testing. Evaluation of two non-overlapping *MED12*-targeting siRNAs in OCI-AML3 cells included both transcript and viability readouts, while replicated TLNP-based experiments in OCI-AML3 and THP-1 demonstrated reductions in *MED12* transcript abundance and cell viability. TLNP-siMED12 was also associated with prolonged survival in the systemic OCI-AML3 model. Hematopoietic selectivity, receptor-dependent delivery and *in vivo* target engagement remain to be established.

## Data Availability

The original contributions presented in the study are included in the article/Supplementary Material, further inquiries can be directed to the corresponding authors.
