# Supplementary material for: Functional-genomic prioritization identifies MED12 as a candidate RNAi vulnerability in acute myeloid leukemia: evaluation of a T22 peptide-functionalized LNP-siRNA formulation
Source: Front Pharmacol. 2026 Sep 23;17:1923515. doi: 10.3389/fphar.2026.1923515 (PMC13642598; doi:10.3389/fphar.2026.1923515)
Supplement: Supplementary file 1 [file Supplementaryfile1.docx]

**Supplementary Figures**

**Supplementary Figure S1**. Evidence provenance and archived scoring framework for AML RNAi-candidate prioritization. **(A)** Public-data inputs, annotated model counts and metric-specific denominators used for the primary DepMap Public 26Q1 ranking and the DepMap 25Q3 robustness comparison. **(B)** Provenance, direction and score range of data-derived components, project-derived numerical components and risk-proxy penalties. **(C)** Archived construction of the exploratory integrated score, including component formulas and penalty terms. **(D)** Candidate ordering and direct experimental advancement of *MED12*. Missing values were assigned 0 for the corresponding component; this convention indicates no applied contribution or penalty.

**Supplementary Figure S2.** Exploratory *MED12* mechanism-context analyses in BeatAML. **(A)** Curated genes showing positive or negative Spearman correlations with *MED12* expression. **(B)** Selected pathway-enrichment signals from the *MED12*-high versus *MED12*-low comparison; bubble size represents the odds ratio and color denotes the direction of enrichment. These associations are hypothesis-generating and do not establish direct *MED12* regulation or a causal downstream mechanism.

**Supplementary Figure S3.** Clinical/subtype and drug-response context for *MED12* in BeatAML. **(A)** *MED12* expression summarized across ELN 2017 risk categories and *FLT3*-ITD and *NPM1* status. **(B)** Spearman associations between *MED12* expression and ex vivo drug-response area under the curve for selected compounds. These analyses are exploratory and do not establish *MED12* as a predictive biomarker or indicate direct pharmacological targeting of *MED12*.

**Supplementary Figure S4.** Lipofectamine 2000-mediated evaluation of two non-overlapping MED12-targeting siRNAs in OCI-AML3 cells. OCI-AML3 cells were transfected with siScramble, siMED12-1 or siMED12-2 using Lipofectamine 2000; PBS was included as a reference control. (A) RT-qPCR analysis of relative MED12 mRNA expression. siMED12-1 reduced MED12 mRNA relative to siScramble (P < 0.0001) and produced a greater reduction than siMED12-2 (P = 0.0084). (B) CCK-8 analysis of cell viability. siMED12-1 reduced cell viability relative to siScramble (P < 0.0001) and produced a greater reduction than siMED12-2 (P = 0.0011); siMED12-2 also showed lower mean viability than siScramble. Data are presented as mean ± SD from three independently initiated biological experiments. Each experiment comprised three technical wells that were averaged before analysis, and individual points represent independent biological experiments. The indicated pairwise comparisons were analyzed using unpaired two-tailed t-tests. PBS was displayed as a reference control and was not included in the inferential comparisons.

**Supplementary Figure S5.** Short-term stability and qualitative siRNA-release observations for TLNP-siRNA. **(A,B)** Particle size and PDI were monitored during storage at 4 °C. **(C,D)** Particle size and PDI were monitored after incubation in DMEM containing 10% (v/v) FBS at 37 °C. **(E)** For the qualitative release condition, TLNP-siRNA was incubated with 1% (v/v) Triton X-100 for 30 min at room temperature before electrophoresis. Individual points represent technical measurements from a single formulation batch and do not represent independently prepared batches.

**Supplementary Figure S6.** Replicated THP-1 validation of TLNP-siMED12. **(A)** RT-qPCR and **(B)** CCK-8 outcomes for PBS, TLNP-siScramble and TLNP-siMED12 are mean ± SD from three independently initiated biological experiments. Each experiment comprised three technical wells that were averaged before analysis. TLNP-siScramble and TLNP-siMED12 were compared using an unpaired t-test in GraphPad Prism version 8.3.0. For panel A, statistical analysis was performed on 2^−ΔΔCt^ relative-expression values. PBS was displayed as a reference control and was not included in the inferential comparison. The corresponding P values were P = 0.0048 for RT-qPCR and P < 0.0001 for CCK-8. Group labels are i, PBS; ii, TLNP-siScramble; and iii, TLNP-siMED12. These results provide replicated validation in a second *MED12*-dependent AML model.
